# The Culture is Prevention Project: measuring cultural connectedness and providing evidence that culture is a social determinant of health for Native Americans

**DOI:** 10.1186/s12889-023-15587-x

**Published:** 2023-04-21

**Authors:** Paul Masotti, John Dennem, Karina Bañuelos, Cheyenne Seneca, Gloryanna Valerio-Leonce, Christina Tlatilpa Inong, Janet King

**Affiliations:** 1 Native American Health Center, Community Wellness Department, 3124 International Blvd., Oakland, CA 94601 USA; 2 Claremont Graduate School Applied Social Psychology Health and Prevention Lab, 150 E. 10Th Street, Claremont, CA 91711 USA; 3 The California Indian Museum and Cultural Center, 5250 Aero Dr, Santa Rosa, CA 95403 USA

**Keywords:** Native American, Indigenous, Culture, Mental health, Physical health, Determinant of health, Prevention

## Abstract

**Background:**

It is important for non-Native persons to understand that the meaning of culture to Native American/Indigenous Peoples is not about esteem, taste or music but rather is described as a cognitive map on how to be. Native American/Indigenous culture can be thought of as all the things and ways in which Native/Indigenous people understand who they are, where they come from and how they are to interact with others. Hundreds of years across many generations have taught that culture-based activities and interventions improve Native/Indigenous health and wellbeing. We explore if increased Native American culture/cultural connectedness is associated with better mental health/well-being and physical health.

**Methods:**

We analyzed data from a two-phased study (*N* = 259 and *N* = 102) of 361 urban Native Americans in California (2018–2021). The 29 items validated Cultural Connectedness Scale-California (CCS-CA) measured Native culture/cultural connectedness. Mental health/well-being and physical health were assessed using the: modified Herth Hope Index (mHHI), Satisfaction with Life (SWL), Center for Epidemiologic Studies Depression Scale-Revised (CESD-R-10), Substance Abuse (CAGE-AID), and Health Related Quality of Life (HRQOL). We conducted Pearson correlations and stepwise regression analyses with CCS-CA as the independent (predictor) variable to explore our main research questions: 1) Is increased Native American/Indigenous culture associated with: 1) better mental health/well-being; and 2) better physical health?

**Results:**

Increased Native/Indigenous culture (CCS-CA scores) is significantly associated with better mental health/well-being (mHHI, *p* < .001) and satisfaction with life (SWL, *p* < .001) predicts good physical health days (HRQOL, *p* < .001). Increased connection to Native American/Indigenous culture (CCS-CA scores) is significantly associated with decreased risk for depression (CESD-R-10, *p* < .0) and substance abuse and (CAGE-AID, *p* < .07). Significant results for culture as protective against risk for substance abuse (CAGE-AID) was most likely affected (*p* value approaching significance) due to an error in language on the measure (i.e., created double negative).

**Conclusions:**

Native American/Indigenous culture is a predictor of improved outcomes for mental health/well-being and physical healthy days. Native culture is an important social determinant of health. We add to the evidence that Native/Indigenous culture (i.e., cultural connectedness) be considered an important intervention objective and health-related outcome measure.

## Introduction

This paper describes results from the multi-phased Culture is Prevention Project which has a primary aim of exploring the link between Native American/Indigenous culture and health outcomes. Enshrined in the Culture is Prevention project is the recognition that for Native Americans/Indigenous Peoples, culture is a social determinant of spiritual, mental, emotional, physical and community health.

Prior to colonization, Native Americans across North America maintained health and wellness since time immemorial through culturally based practices. According to Indigenous worldviews, the environment, mind, body, and emotional health are inextricably linked to collective human behavior, practices, wholeness, and, hence, wellness [1, 2]. Unfortunately, colonization and government policy had a devastating impact on eroding Native culture resulting in the subsequent impact on ill health [3, 4]. Examples include the removal from traditional lands, loss of ways of life, disruption of the practice of culture, removing children from homes for involuntary attendance at Boarding Schools where they were punished for speaking their languages or ‘behaving Native’ and removing Native children from their families via our Child Welfare system and making it illegal to practice Native culture [3–6]. Health and social consequences of colonization include: i) multi-generational trauma; ii) loss of land and culture; iii) high prevalence rates for chronic disease, suicide, and substance abuse; and iv) poor social outcomes such as homelessness, unemployment, family violence, and incarceration [7–13]. Historical and ongoing impacts of colonization continue to plague Indian Country in ways that aim to break apart Native communities from cultural practices.

### What is the meaning of Culture to Native Americans and why is this important for Non-Native persons to understand?

The meaning of culture to Native Americans is an imperative foundation that non-Native people need to understand, and is well described by Seneca (2020):

“*Culture for Native Americans is not about esteem, taste, or music but rather a cognitive map on how to be. Culture can be thought of as all the things and ways in which Native people understand who they are, where they come from and how they are to interact with others. Native Americans learn these principles including beliefs, values, and behavior from traditional stories, ceremony and language instructed by family and community.*

*It is important for Native people to engage with core elements of culture (e.g., creation stories/mythology, ceremony, and language) because it promotes intergenerational transmission of historical and traditional knowledge, positive identity development of youth, and a strengthening of social ties within families (i.e., interdependence). Hundreds of years across many generations have taught us that culture-based activities & interventions improve our health & wellbeing* [14].”

### Indigenous approaches to assessing cultural connectedness, physical health and mental health/well-being

Native American/Indigenous peoples have known for generations that health is embedded in their cultures. This means for Native/Indigenous peoples, that culture is determinant of health, and that loss of culture is a risk factor; whereas strengthening, reconnecting or reclaiming is protective on multiple levels. The extant research has provided strong evidence supporting the above assertion. Chandler and LaLonde [15], in a 5-year multi-community study (*N* = 196 communities) of 2,495 Native youth suicides in Canada, evaluated the relationships between cultural continuity, self-continuity, and local communities’ initiatives at ‘cultural rehabilitation’ (i.e., reclaiming and indigenizing) that included: i) self-government, ii) land claims, iii) education, iv) health services, v) police & fire protection, vi) implementing ‘cultural facilities’. Conclusions included: a) communities that initiated changes to rehabilitate their cultures had dramatically lower suicide rates; and b) communities that did not initiate any of the six protective steps had suicide rates 5 to 100 times the provincial average [15].

Studies also demonstrate an expanding need in developing and using Native/Indigenous approaches to conceptualize community health [16] and also in approaches to assessing cultural connectedness and the link to health outcomes (i.e., Indigenizing health research). For example, Snowshoe et al., developed the Cultural Connectedness Scale (29 items) and Cultural Connectedness Scale-Short (10 items) to demonstrate the important relationships between culture and mental health/well-being [5, 13]. Peters et al., developed the Wicozani instrument (9 items) as an Indigenous measure of ‘overall health and well-being’ [17], and Walls et al., developed a ‘social-cultural integration’ measure [18]. In addition to these, King et al., (2019) and Masotti et al., (2020) adapted and validated the Canadian Cultural Connectedness Scale for use in an urban dwelling diasporic Native/Indigenous California community [19, 20].

These studies and other research provide support and evidence towards trends in the development of Native/Indigenous, holistic, strength-based approaches that are embedded in Native/Indigenous epistemological approaches versus using the Western model’s more deficit/risk based approach. This paradigm and model shift, has support and is being argued by multiple researchers to move away from interventions and measurement tools solely created from western paradigms (See, [13–21]). With this paper we provide further support to continue this approach. Our findings further support the assertion that ‘culture is prevention’ and also establish the for the first time, in the Culture is Prevention project using the CCS-CA instrument, the link between Cultural Connectedness and physical health.

### What is the Culture is Prevention Project and why was it started?

The Culture is Prevention Project was initiated by the Community Advisory Workgroup, comprised of six Urban Indian Health Organizations in the San Francisco Bay area. This was in response to issues that emerged in projects funded by the SAMHSA and the California Department of Public Health’s innovative California Reducing Disparities Project.

Issues identified included: i) the lack of culturally informed methods to evaluate, from an Indigenous/Native perspective, the positive outcomes of culture-based programs to improve health and well-being; and ii) interest in providing an approach that recognized the relationship between Native American/Indigenous culture and health [19, 20]. The Culture is Prevention Project evolved into a 6-phased community-based participatory research (CBPR) program (See Table 1).

**Table 1:** Culture is Prevention Project

Phase 1	Consensus Generating Workshop
Phase 2	Literature Search & Knowledge Synthesis
Phase 3	Adapting the Snowshoe Cultural Connectedness Scale (CCS) for Multi-Tribal Communities in California
Phase 4	Pilot Testing/Validation of the Cultural Connectedness Scale – California (CCS-CA) and Evaluation of the Relationship between Culture and Mental Health/Well-being
Phase 5	Exploring the Predictive Properties of the CCS-CA
Phase 6	Cultural Connectivity, Integration, Health (Physical/Mental), & Health Services Utilization

Phases 1–2 were presented in a 2019 paper [20] where the most notable results were the identification of the original Cultural Connectedness Scale (CCS) developed by Dr. Angela Snowshoe, an Indigenous scholar in Canada. Snowshoe developed the CCS to measure the degree of cultural connectedness with the objective of demonstrating the link between Indigenous culture as an important protective factor for health, resiliency, and well-being [5]. In Phase 3, we worked with six Urban Indian Health Organizations to adapt the CCS to be appropriate for the multi-tribal diasporic communities in California. The result was what we refer to as the Cultural Connectedness Scale-California (CCS-CA). Following this, and in a 2020 paper, we report the results from Phase 4 where we validated the CCS-CA in a sample of Native adults and where we replicated Dr. Snowshoe’s results that increased culture was associated with better mental health and well-being [5, 14].

We report our findings from the Culture is Prevention research program that includes components of Phases 5 and 6. COVID-19 deeply impacted the participation capacity of our partnering organizations thus causing delays in data gathering and requiring methodological changes. However, we believe the results and implications are robust, notable, and interesting.

## Methods

### Design (COVID impact)

This project was funded by Blue Shield of California Foundation and originally had a different data collection design, timeline and sample size. This was originally two different studies with study one in the San Francisco Bay Area and the study two in other urban areas (e.g. Sacramento, Fresno). We began collecting data for the original study and then COVID negatively impacted the project. We had to interrupt the original study, modify its design, and move all measures onto Qualtrics. At that time some measures, that under the original design would have been included in study two and would have been analyzed in two separate studies. were now going to be included in a two-step single study. We had to do a small pilot study to ensure accuracy of all measures that were moved online that were not used in step one.

We had to limit the participation from other sites in the project due to remoteness and online access limitations without access to direct support. The data was then analyzed separately for step one and then step two. We then aggregated data on instruments that were both in step one and step two and report them here (See Table 4).

### Participants

Our participants ($\sim$ 300 adults) identified as Urban Dwelling Native American (and/or Indigenous, First Nations or American Indian). The original study design (i.e., prior to COVID-19) was to conduct a two-step study where Steps 1–2 shared a ‘baseline’ grouping of instruments and in Step two we added several instruments to specifically measure risk for depression and substance abuse.

#### Step one (2018– 2019, *N* = 259)

Step one focused on diasporic Urban Dwelling Native American adults in the San Francisco Bay area. Over three hundred agreed to participate. However, after review and data cleaning, 259 full instrument packages were appropriate for analyses. Participant recruitment employed mixed methods: a) invitations to clinic patients who identified as Native American/Indigenous and who received services in the previous 18 months; b) convenience samples at cultural events; c) Community Advisory Board that agreed to identify community members; and d) referrals from clinic staff and community leaders. Step one participants completed paper versions of the instrument packages.

#### Step two (2019–2021, *N* = 102)

Step two was intended to include a sample from among nine Urban Indian Health Organizations (UIHOs) affiliated with the California Consortium for Urban Indian Health. Unfortunately, COVID-19 decreased the UIHOs capacity to participate. The final sample (*N* = 102 versus 205 planned) encompassed San Francisco Bay area, North to Santa Rosa and Southeast to Fresno. To adjust for COVID, we implemented the use of Qualtrics as a virtual data collection system. Table 2 provides descriptive statistics for the participants.

**Table 2:** Participant descriptive statistics

	Step 1	Step 2
Participants	*N* = 259	*N* = 102
Age (Range, Mean, *SD*)	(18–84, 45.1, *17.57*)	(18–79, 41.8, *16.4*)
Gender Identity		
Female	157	75
Male	78	25
Transgender	2	1
Two Spirit	13	0
Gender Queer/Non-Conforming	2	1
Did Not Identify	1	0
Tribal Affiliations (total separate Tribes = 105)		
One	194	69
Two	44	22
Three	12	6
Four or More	9	5

## Research questions and hypotheses

### Research questions

1. In Adult Native Americans living in urban California, does the CCS-CA and its subscales link levels of cultural connectedness to levels of hope and satisfaction with life (measures of eudemonic wellbeing) as measured by the mHHI and SWLS?
2. In Adult Native Americans living in urban California, does the CCS-CA and its subscales link levels of cultural connectedness to those experiencing or at risk for depression as measured by the CESD-R-10?
3. In Adult Native Americans living in urban California, does the CCS-CA and its subscales link levels of cultural connectedness to those experiencing or at risk of substance/alcohol abuse as measured by the CAGE-AID?
4. In Adult Native Americans living in urban California, does the CCS-CA link levels of cultural connectedness to those experiencing or at risk of domestic violence as measured by the Perception of Domestic Violence-Short Form?
5. In Adult Native Americans living in urban California, does the CCS-CA link levels of cultural connectedness to levels of physical health (i.e., number of good health days) as measured by the HRQOL-14?

### Hypotheses

1. The CCS-CA will be positively correlated and a significant predictor of mHHI and SWLS (Eudemonic Wellbeing) indicating culture is a social determinant of health.
2. The CCS-CA will be negatively correlated and a significant predictor of substance/alcohol abuse, and depression. As measured by the CAGE-AID and CESD-R.
3. The CCS-CA will be negatively correlated and a significant predictor of perception of domestic violence (a proxy for DV risk).
4. The CCS-CA will be a significant predictor of physical health (i.e., number good health days) as measured by the HRQOL-14.

### Measures

Except for the Perceptions of Domestic Violence instrument (which we discontinued use), the measures used had good reliability with Cronbach alphas > 0.60 (See Table 3).

**Table 3:** Reliability of measures

Measure	Cronbach alpha	Confidence Interval 95%
CCS-CA	0.93^b^	(0.86,0.94)
mHHI	0.83^b^	(0.82,0.95)
SWL	0.9^b^	(0.86,0.93)
CESD-R	0.82	(0.77,0.86)
PDV	0.43^a^	(0.86,0.94)
HRQOL	0.70	(0.66,0.81)

^a^PDV was not included in any analysis as it was not a reliable measure

^b^Combined data used

#### Cultural connectedness scale-California (CCS-CA)

The CCS-CA is a 29-item validated instrument that measures the Native American/Indigenous culture/cultural connectedness on three subscales: i) Identity, ii) Spirituality, and iii) Traditions [5, 14, 19]. (The CCS-CA was adapted from the original CCS developed by Dr. Angela Snowshoe in Canada.)

#### Health related quality of life-14

The CDC HRQOL-14 contains fourteen items to measure health-related quality of life. The items are designed to measure broad influences on life, including distal social and environmental factors such as housing, income, social support, and access to care [22, 23].

#### Modified herth hope index

The modified Herth Hope Index (mHHI), is a 12-item instrument with good psychometric properties and has been validated in multiple populations. Hope serves as a proxy measure for mental health and well-being. Hope is known to positively influence the onset, duration, prognosis, and recovery from mental and physical illnesses [24–28].

#### Satisfaction with life scale

The SWLS is a 5-item instrument designed to measure global cognitive judgments of satisfaction with one's life and well-being. Scores on the SWLS have been shown to correlate with measures of mental health and to be predictive of future behaviors such as suicide attempts [29].

#### Perception of domestic violence-short version

This 10-item instrument assesses perception of violence between intimate partners. Scenarios presented are considered examples of domestic violence with each scenario rated on a Likert scale: 1 = never, 2 = sometimes, 3 = often, and 4 = always [30]. We discontinued and do not report use of this instrument because results indicated a Cronbach alpha (α = 0.43).

#### Center for epidemiologic studies—depression scale (CESD-R-10)

The CESD-R-10 scale is a brief self-report scale that measures self-reported symptoms associated with depression experienced in the past week. The CESD-R-10 has been shown to be a reliable measure for assessing the number, types, and duration of depressive symptoms across racial, gender, and age categories [31–33].

#### CAGE-AID

The CAGE-AID is questionnaire where the focus of each item on the original CAGE was expanded from alcohol alone to include alcohol and drugs. This allows for the concurrent screening of substance abuse and dependence issues (e.g., alcohol and drugs) [34].

## Results

### Overview

We found support for four of the five research questions. The CCS-CA and its subscales (i.e., Culture/Cultural Connectedness: i] Identity, ii] Spirituality, & iii] Traditions) are linked to eudemonic wellbeing as measured by hope and satisfaction with life. In Step two the CCS-CA is linked with depression as measured by the CESD-R (*p* < 0.001), and although risk for substance use and abuse approached significance as measured by CAGE-AID (*p* = 0.078) it is not reported here, but the reason for this finding is discussed. The perception of domestic violence measure did not achieve reliability criteria during Step one (α = 0.43) and is not reported here as we were unable to answer research question four. Finally, we established a link between CCS-CA and number of good health days as measured by the HRQOL-14.

The CCS-CA and its subscales has been established as a valid and reliable measure of Cultural Connectedness in this population with Cronbach Alpha range α = 0.87 thru 0.92 across several studies [5, 14, 19]. We found support for all hypotheses except Hypothesis three. Hypothesis one was supported and establishes the CCS-CA and its subscales with links to Hope and Satisfaction with life (i.e., subscale Identity predicted Hope while Identity and Spirituality predicted Satisfaction with Life). Hypothesis two was supported in Step one with CCS-CA being negatively correlated and a significant predictor of substance/alcohol use and abuse as well as depression. Finally, Hypothesis four was supported with CCS-CA being the most significant positively correlated predictor of number of good physical health days in the past 30 days.

We suspect the Perception of Domestic violence measure may not be an appropriate measure for this population. Our findings provide ongoing support that the CCS-CA is a significant predictor of eudemonic wellbeing, risk for substance use and abuse as well as depression, and number of good physical health days. With this study and our previous studies [14, 19], it is putative with robust findings and support that Native culture is a social determinate of health, eudemonic wellbeing, and a protective factor in this population.

#### Results step one study

This was a correlational and regression analysis design. Initially data was collected (*N* = 300)*.* After reviewing the instruments for completion and data cleaning the final sample size (*N* = 259).

We alternated the order of the step one instruments to evaluate if the order affected responses. We had three different ordered packets that were randomly handed to participants. Verbal and written consent were obtained from participants. We found that there was no order effect of the CCS-CA. F (2, 256) – 0.566, (p = *ns*).

We did regression analysis with CCS-CA (i.e., culture measure) as the independent variable (predictor) and mHHI as the dependent variable (i.e., mental health/well-being measure). We found CCS-CA was a significant predictor of mHHI *F* (1, 257) = 10.74 (*p* < 0.001) *B* = 0.20 with *R*^2^ = 0.04. We did a regression analysis with CCS-CA as the independent variable (predictor) and SWL (i.e., Satisfaction with Life) as the dependent variable. We found CCS-CA was a significant predictor of SWL *F* (1, 257) = 25.79 (*p* < *0.001)*, *B* = 0.30 with *R*^2^ = 0.09. This supported Hypothesis 1 with CCS-CA overall score was predictive of mHHI and SWL. We did not evaluate Hypothesis 3 as the DVP (i.e., Perceptions of Domestic Violence) did not meet minimum reliability for this population (See Table 3).

We then evaluated predictiveness of CCS-CA three subscales (i.e., Traditions, Identity, Spirituality). We ran regression analysis of all three subscales as the IV (predictor) and the mHHI (i.e., measure of mental health/well-being) as the DV (dependent). We found CCS-CA subscales Traditions and Spirituality did not predict mHHI (*p* = *ns).* We found the CCS-CA subscale Identity significantly predicted mHHI *F* (3, 255) = 7.45, (*p* < 0.001), with *R*^2^ = 0.18. We also ran regression analysis of all three subscales as IV (predictor) and SWL as the DV. We found the CCS-CA subscale Traditions did not significantly predict SWL (*p* = *ns)* CCS-CA subscales Identity significantly predicted SWL F (3, 255) = 9.82, (*p* <  < 0.002), with *R*^2^ = 0.29 and Spirituality *F* (3, 255) = 8.757, (*p* < 0.001), with *R*^2^ = 0.19. This indicates that although these subscales were highly correlated and had some shared variance [19] they are useful subscales and distinct subscales within the CCS-CA and in predicting different outcomes.

These findings partially supported other hypotheses in that traditions and identity subscale did not significantly predict mHHI. However, spirituality and identity subscales did significantly predict SWL. This also establishes that although the three subscales are highly correlated, they are separate constructs with different predictions as it relates to Hope and Satisfaction with Life scales. Overall, H1 was supported with CCS-CA (i.e., culture) being positively associated with both mHHI and SWL (i.e., mental health & well-being). H2 was supported as the CCS-CA was negatively correlated with risk of substance abuse and depression. H3 was not answered as the Domestic Violence measure did not reach the traditional minimal threshold for reliability. Finally, H4 was supported as the CCS-CA was a significant predictor of good physical health days.

#### Results step two study

This was completed after the measures were migrated onto an online format. We piloted the measures to ensure that they were correctly loaded. We set the program to alternate order of measures between subjects. The measures were loaded onto the Qualtrics platform (an online data collection program) (*N* = 127). After reviewing the instruments for completion and data cleaning the final sample size (*N* = 102). The Chi-Square goodness of fit was not significantly different between Step one data and Step two data for each of the scales and demographics.

Regression analysis of step two data found similar results to Step one data in that the CCS-CA was a significant predictor of Hope *F* (1,101) = 15.03, *p* < 0.001 (*R*^2^ = 0.13). The CCS-CA was also a significant predictor of Satisfaction with Life *F* (1,101) = 11.05, *p* < 0.001 (*R*^2^ = 0.093). The CCS-CA was a significant predictor of Depression *F* (1,101) = 6.26, *p* < 0.01 (*R*^2^ = 0.06).

Additionally, we included in Step two the CAGE-AID substance use screen to (Yes or No “I have used drugs or alcohol in the past 12 months). An independent sample *t-test* was not significant *t* (1) = 1.36, *p* = 0.078. This non-significant finding may have been affected by the wording of the questions when moved to Qualtrics. The wording created a double negative and caused confusion. We ask about the number of bad days (Physical Health) on the Health-Related Quality of Life (HQROL) and then subtracted the number of bad days from 30 to establish the number of good days experience in the last 30 days taking a strength-based approach *M* = 20.09. We performed a regression analysis of the CCS-CA to predict the number of good days. The CCS-CA (i.e., culture measure) was a significant predictor of Good Days, *F* (1, 101) = 12.75, *p* < 001. We include descriptive data (i.e., *Mean* and *Standard Deviations*) for steps 1 and 2 as well as the combined data (See Table 4).

**Table 4:** Step one and step two with combined data descriptives

	**Step 1**		**Step 2**		**Combined**	
**Variables**	***N* = 259**		***N* = 102**		***N* = 361**	
	*M*	*SD*	*M*	*SD*	*M*	*SD*
CCS-CA	4.21	.61	3.77	.60	4.10	.56
Herth Hope Index (modified) mHHI^a^	3.99	.55	3.91	.58	3.97	.56
Satisfaction with Life (SWL)	4.85	1.30	4.66	1.56	4.80	1.38
Perceptions of Domestic Violence PDV^b^	2.27	1.11	^c^	^c^	^c^	^c^
Center of Epidemiology Studies Depression Scale (CES-D)	^c^	^c^	2.04	.58	^c^	^c^
Historical Loss Scale (HLS)	^c^	^c^	3.47	1.18	^c^	^c^
Health Related Quality of Life (HQROL)	^c^	^c^	20.09	9.25	^c^	^c^

^**a**^Herth Hope Index was modified with permission from Dr. Herth to include (Neither/Nor)

^b^PDV not included in analysis

^c^Intentionally left blank

#### Aggregated data

There were differences between Step one Study and Step two Study collected data and measures. When we combine the data, we were only able to combine data on measures that were in both Step one and Step two. We did not collect data on the Domestic Violence Perception as it proved to be unreliable Cronbach (*a* = 0.43) We then added the Depression scale (CES-D) to establish divergent validity and directly linking CCS-CA and Depression. We found there to be a significant negative correlation *p* < 0.05. This was also in line with the negative correlation between the Satisfaction with Life Scale and Depression *p* < 0.01 (See Table 5).

**Table 5:** Bivariate correlations combined step one and step two

	1	2	3	4	5
1. Cultural Connectedness Scale (CCS-CA)	-				
2. Herth Hope Index (modified) (mHHI)	.36^b^	-			
3. Satisfaction With Life Scale (SWLS)	.32^b^	.32^b^	-		
4. Center Epidemiology Study Depression (CES-D)	-.23^a^	-.15	-.64^b^	-	
5. HRQOL No. Good Health Days	.13^b^	.25^b^	.33^b^	.20^b^	-

^a^ Correlation is significant at < .01

^b^ Correlation is significant at < .001

An analysis of the individual outcome variables and the predictor variable of CCS-CA was significant. The CCS-CA and Hope mHHI *F* (1,359) = 15.03, *p* < 0.001. As expected, the variance accounted for increased monotonically as more variables were included. Throughout step one, step two and the aggregated data the single model predictor with the overall CCS-CA score being the best fit (*ps* < 0.001).

## Discussion and public health implications

### Native/indigenous culture is an important social – determinant of health

This is the third large sample study using the Cultural Connectedness Scale in Canada (CCS *n* = 319) and CCS-CA in California (*n* = 344 and this study *n* = 361) where all demonstrated that increases in Native/Indigenous culture is associated with better eudemonic well-being [5, 19]. Results from this project add to the evidence supporting the assertion that for Native Americans/Indigenous Peoples, traditional culture is an important social determinant of health [5, 13, 14, 19]. In addition, to our knowledge, this is the first study, that links Native/Indigenous Culture (measured by CCS-CA) with better physical health. This finding aligns with what Native Peoples have known for millennia which is that health is embedded in their culture.

### Prevention and a need for a healthcare paradigm shift

Health for Native/Indigenous Peoples has been negatively impacted as a result of years of colonization and government policies that resulted in loss of culture and ways of life [4, 6, 13–19]. We know that Indigenous Peoples experience higher rates of morbidity and mortality and that the Western medical model and government responses, intended to address health and social disparities, have not been effective.

Given that the loss of culture has negatively impacted the health and well-being of Indigenous Peoples, we argue that the degree of reclaimed traditional culture or increased cultural connectedness is an important health-related outcome measure which at times may be more important than the reduction in frequency of some risky behaviors or risk factors commonly the focus of Western modalities. In addition, we argue that if government and healthcare is committed to improving Native American/Indigenous health, they should become cognizant that Indigenous cultures historically manufactured good health. All should try to better understand, learn from Indigenous epistemology and approaches to health/healing and support integration into the healthcare system. We argue that doing so will increase overall healthcare system capacity and serve to promote health equity and social justice.

To support the above assertion, we refer to our ongoing study of Native American adults in California (*n* = 500), where 87% want increased access to traditional healers and spiritual leaders in healthcare. Additionally, 44% percent indicated they delayed care or did not want to return for healthcare services due to negative experiences with the medical professional (or staff) not understanding or respecting their culture or language. These results provide evidence indicating a possible contributing factor to community health disparities—as delayed access to medically necessary care is associated with increased, pain, suffering, mental anguish, deteriorations of current conditions, and mortality. Delay can also have high economic consequences such as work absenteeism, and decreased productivity or ability to work [35, 36].

### Evaluating properties of the CCS

In Appendix B in our first paper [19], we provide directions on how the CCS-CA could be adapted for other Native American/Indigenous communities which would support future use and research. With more research, the twenty-nine item Cultural Connectedness Scale, with its three subscales (Identity, Spirituality & Traditions) has the potential to screen for strengths and risks associated with health (e.g., mental, physical, and domestic health). This research supports this potential. However, we feel it needs more research and consultations with Native American/Indigenous traditional healers. This could then guide health and healing prescriptions. In addition, the CCS-CA may be used in logic models when evaluating programs and their efficacy. Increased Cultural Connectedness can be a short-term, mid-term, or outcome measure based on culturally based interventions, program protocols and implementations.

### Native domestic wellbeing/violence

There is an interest in developing a culturally driven Native/Indigenous instrument that focuses on measuring domestic well-being and identifying risk for domestic violence. The Native ‘strength-based’ approach to family health supports the assertion that: “Native culture and values are incompatible with domestic violence.” An Indigenous-informed and developed instrument that can inform/guide domestic well-being and identity risk for domestic violence could then function as a therapeutic intervention.

## Conclusion

Through this research we provide further evidence for a paradigm shift away from the long-held belief and deficit based models that Native Identity and Native Culture are linked with the development of health disparities. Walters and Simoni [21], proposed that Indigenous Identity and Culture could function as a buffer against these developments. Multiple researchers provide support for a paradigm shift (See, [13–20]). We have convergent support that higher levels of cultural connectedness are linked to higher levels of hope and satisfaction with life. We also have provided divergent support in that higher levels of cultural connectedness are linked to lower levels of depression. In the future researchers when designing programs of research, prevention or intervention should consider this strength-based resilience-based approach in that the strengthening of Native/Indigenous Cultural Connectedness can have an overall positive influence on mental and physical health outcomes for Native/Indigenous people. That Western Psychological and Medical Models need to be cognizant and consider developing and implementing Native/Indigenous culturally informed community evidence-based practices that strengthen cultures and aids in cultural reclamation [15].

## Data Availability

Summary information based upon the data from this study is available from the corresponding authors. However, since this is a CBPR project, all community-level data is owned by the participating Native American/Indigenous communities and could not be made available by the authors without explicit permission from the participating communities.

## Abbreviations

CAGE-AID: CAGE-Adapted To Include Drugs
CBPR: Community-based Participatory Research
CESD-R: Center for Epidemiologic Studies Depression Scale-Revised
CCS-CA: Cultural Connectedness Scale-California
CCS: Cultural Connectedness Scale
HRQOL: Health Related Quality of Life
mHHI: modified Herth Hope Index
PDV: Perception of Domestic Violence
SWLS: Satisfaction With Life Scale
SAMHSA: Substance Abuse and Mental Health Administration
UIHO: Urban Indian Health Organizations
